# Decoding multicellular interaction networks–a new horizon in tumor microenvironment research

**DOI:** 10.1002/1878-0261.13810

**Published:** 2025-02-05

**Authors:** Roi Balaban, Merav Cohen

**Affiliations:** ^1^ Department of Clinical Microbiology and Immunology, Faculty of Medical and Health Sciences Tel Aviv University Israel

**Keywords:** cell–cell communication, personalized medicine, PIC‐seq, scRNA‐seq, spatial niches, tumor microenvironment

## Abstract

The tumor microenvironment (TME) milieu directs a plethora of tumor‐modulating functions. Recent years have seen pivotal breakthroughs in our understanding of the TME's role in tumor initiation and progression, with tangible clinical applications. Individual components of the TME exert their function predominantly by cell–cell crosstalk, both in the form of physical interaction and secreted factors. Notably, different spatial niches represent exclusive signaling hubs in the TME, propagating pro‐ or antitumoral functions. The exploration of these interactions has been vastly facilitated by novel molecular technologies, each of which provides a different perspective on this intricate intercellular communication network. Together, these complementary methods paint a detailed, high‐resolution map of the TME's interaction landscape. In this viewpoint, we explore how cellular interactions can unlock a new level of understanding of TME complexity, and discuss the promises and challenges of characterizing tumors based on their cellular interaction footprint.

## Graphical abstract

Cells in the tumor microenvironment influence tumor establishment and progression through an elaborate array of cellular interactions. Integrating transcriptomic, spatial, and intercellular crosstalk data at single‐cell resolution unveil complex, functionally distinct multicellular circuits. These may be utilized for patient stratification to improve prognostication and treatment personalization as well as identification of novel potential therapeutic targets.

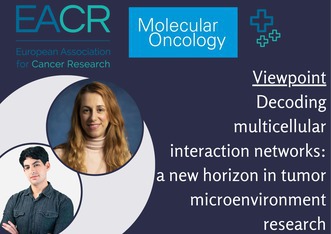

**Abbreviations**
CAF: cancer‐associated fibroblast
ECM: extracellular matrix
PIC‐seq: physically interacting cell sequencing
scRNA‐seq: single‐cell RNA sequencing
TAM: tumor‐associated macrophage
TME: tumor microenvironment
uLIPSTIC: universal labeling of immune partnerships by SorTagging intercellular contacts

The tumor microenvironment (TME) is a heterogeneous compartment, where neoplastic cells reside in close proximity to immune and stromal cells, embedded in an altered extracellular matrix (ECM). In recent years, extensive research into components of the TME has drastically furthered our understanding of their function and importance in tumor establishment, progression, and dissemination. This has been largely supported by technological advances, particularly single‐cell RNA sequencing (scRNA‐seq), which enabled the identification and thorough transcriptomic characterization of rare and distinct cell subtypes and states. Perhaps the most glaring examples of that are tumor‐associated macrophages (TAMs) and cancer‐associated fibroblasts (CAFs). While some tumor‐supporting functions had previously been attributed to those cell types, single‐cell genomics revealed that each of them is comprised of an array of phenotypically distinct subtypes (cell states) carrying out different, sometimes opposing functions within the TME [1, 2]. These newfound insights into the role these cells play in cancer have fueled ongoing attempts to target those specific cell states in the clinic.

While exploring the functional role of individual cell subtypes in the TME has been fruitful, those cells cannot be fully understood outside of the context in which they reside. Indeed, intercellular crosstalk is an important feature of the TME. Cells communicate through an elaborate array of signals delivered by cell–cell physical contact, secreted proteins, and extracellular vesicles. Similar cells residing in different spatial niches are exposed to distinct milieus, dictating the interactions they form [3]. To appreciate just how pivotal those cell–cell interactions are, one needs to look no further than the TAM–tumor cell interaction. Tumor cells are capable of producing chemokines to recruit circulating monocytes, factors that govern the differentiation of those monocytes into macrophages and polarization signals to establish a specific TAM phenotype. The resulting TAM can in turn support the tumor by secreting growth factors, remodeling the ECM, inducing epithelial‐to‐mesenchymal transition and suppressing immune responses [4]. Hence, the tumor‐altering functions of TAMs can largely be attributed to their communication with adjacent cells. Cell–cell crosstalk therefore constitutes another level of complexity to the understanding of the TME.

The research of intercellular crosstalk is largely facilitated by novel technologies that can be used to detect, identify, and molecularly characterize interacting cells. scRNA‐seq data can offer clues in the form of ligand and receptor expression patterns, and multiple computational platforms have been developed to flag potentially interacting cell types based on gene expression alone [5]. Crucially, this approach does not capture *bona fide* interactions, but rather potential pathways through which cells can interact, due to its lack of spatial context. High‐throughput, high‐resolution spatial transcriptomics and proteomics approaches can provide just that with current available platforms approaching single‐cell resolution and improved segmentation [6, 7]. Using these methods, one can characterize cells that reside near each other and identify micro‐niches with distinct cell type compositions and gene expression profiles within the same sample, implying proximity‐based interactions. To investigate how physical interactions shape the cell phenotype, dedicated experimental methods are required. Sequencing of physically interacting cells (PIC‐seq) allows for the transcriptomic characterization of cell doublets as they are interacting, and the elucidation of transcriptomic programs specifically induced by the crosstalk [8]. Similarly, universal labeling of immune partnerships by SorTagging intercellular contacts (uLIPSTIC) is a transgenic mouse model in which cells are specifically labeled for downstream analysis upon physical interaction with a specific cell of interest [9]. These novel approaches can acutely highlight the effects of specific physical interactions; however, they require preselection of the interactions of interest. These and other technologies enable a deeper and more complex understanding of the intercellular crosstalk in the TME.

Each of these approaches provides a different perspective on the architecture of the TME and the cell–cell crosstalk within it, but put together, they paint a picture far more complex. Cell type abundance, transcriptomic phenotype, spatial distribution, and cell–cell communication can all be evaluated simultaneously to map the bidirectional interaction of one cell with its environment. With this type of multilayered snapshot, it is now possible to trace patterns involving multiple cell types and gene programs. One notable example of such elaborate cellular circuits occurs in the liver, where dying Kupffer cells activate local stromal cells to recruit circulating monocytes, which differentiate into new Kupffer cells upon contact with hepatocytes and liver stromal cells [10]. While many cell–cell interactions in the TME have been elucidated [11], the overall complexity of this multi‐component system is yet to be untangled [12]. Utilizing novel tools and integrating multiple approaches holds promise of unlocking this new layer of understanding (Fig. 1A).

**Fig. 1:**
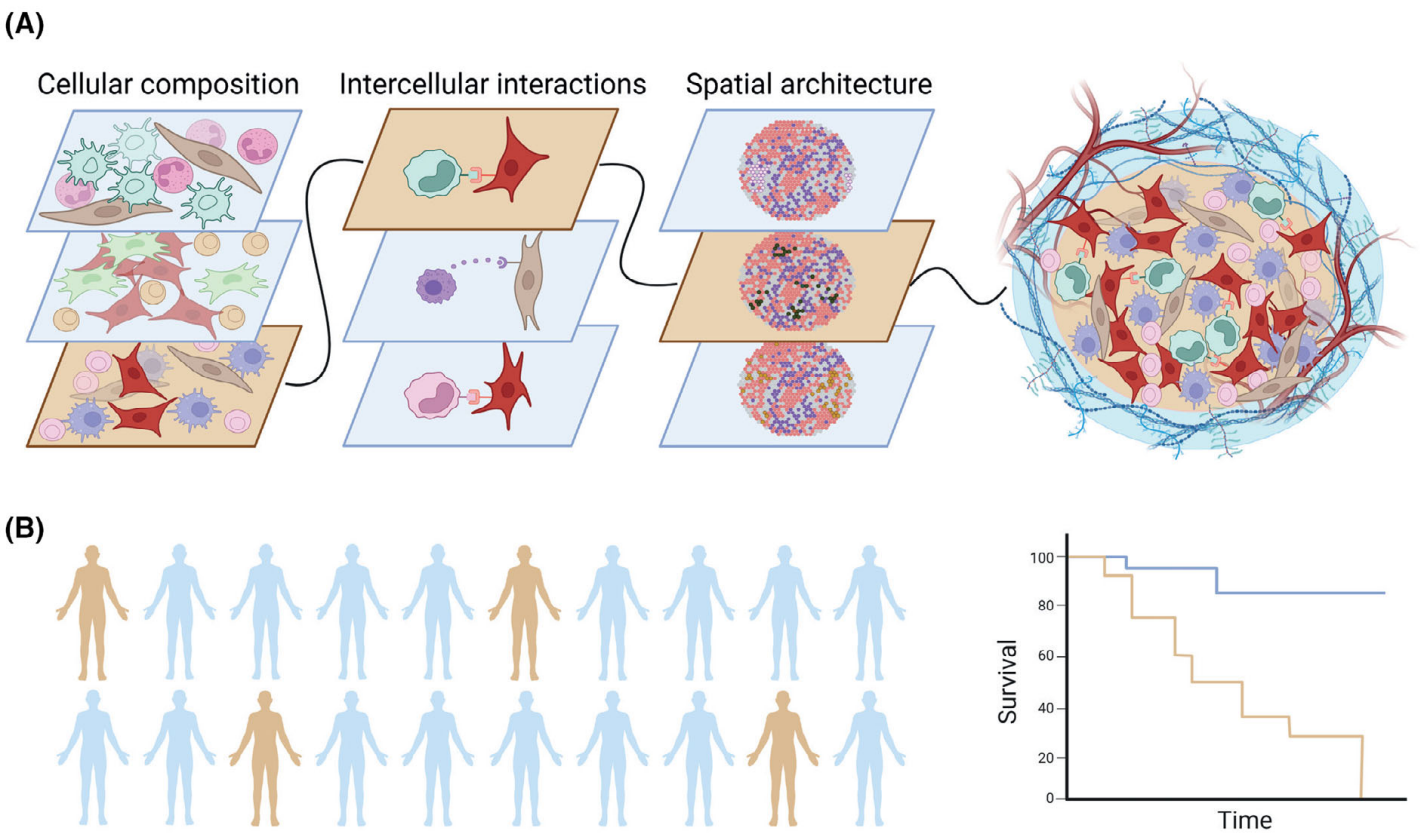
Multimodal analysis of the tumor microenvironment (TME) highlights common patterns associated with clinical features. (A) Characterization of the TME through cellular composition, intercellular crosstalk, and spatial architecture provides a complex understanding of cellular interplay. (B) Common multicellular patterns allow for improved and potentially actionable patient stratification. Created using Biorender.com.

Importantly, methods, such as spatial transcriptomics or PIC‐seq, are better thought of as powerful hypothesis‐generation approaches that can uncover functionally important interactions to be further validated experimentally. Any investigation into the intricate crosstalk between cells is incomplete without functional validation. While each of the aforementioned methods provides novel insights into the cell–cell crosstalk in the TME, none of them is sufficient to prove the effect one cell can have on another. To do this, it is imperative to perturb the interaction and test its direct effect. Such perturbations may range from multicellular *in vitro* co‐culture experiments (including transwell assays validating direct and secreted crosstalk), to *in vivo* targeting of suspected signaling axes.

The challenge of studying a complex multilayered environment becomes even greater when considering the heterogeneity of human tumors. Even when classified by organ and cell of origin, microscopical morphology, stage, genomic alterations, and the expression of key proteins, such as PD‐L1, patients often vary in disease presentation, response to therapy, disease recurrence, and prognosis [13]. Similarly, variability can be observed in the composition of the TME in patients with seemingly similar diseases. This heterogeneity in TME composition makes it challenging to study the function of specific cell types, but exponentially more so when attempting to deconvolute a multicellular network, governed by cellular composition, gene expression, and spatial architecture. To thoroughly understand and convincingly illustrate such a multiparametric system, large high‐quality datasets that integrate multiple complementary modalities are imperative. The generation of such databases is a costly and demanding task which is well on its way thanks to the collaborative efforts of many. As the availability of high‐quality human data continues to increase, it will facilitate the investigation of more complex interactions within the TME, overcoming the challenge of inter‐patient variability.

Heterogeneity in the TME of human tumors constitutes not only a challenge but also an exciting opportunity. PD‐L1 expression on tumor cells varies among patients, and is routinely tested to identify patients who may benefit from immune checkpoint blockade therapy. Similarly, TME immune cell composition of various cancer types can be categorized into one of a few archetypes with correlation to patient prognosis [14, 15]. These examples demonstrate the importance of identifying divergent patterns that may distinguish some patients with common clinically relevant features. Cellular interactions likely behave in a similarly divergent pattern. Even with a similar TME composition, it is possible for tumors to employ different cellular circuits, potentially allowing for further patient stratification and improved treatment selection (Fig. 1B).

The multilayered study of cell circuits within the TME presents significant clinical potential. By characterizing these complex intercellular interactions, we can identify specific interaction patterns that stratify patients based on their unique TME characteristics. This stratification could enable more precise prognostication and predict responses to therapy. Identifying dominant cell circuits in the TME could help define subgroups of patients who may benefit from targeted interventions. Moreover, these insights could unveil novel therapeutic targets, particularly in the form of disrupting key cellular interactions that promote tumor progression. With the advent of novel technologies, and an increasingly complex understanding of the TME, the application of these findings in a clinical setting is becoming tangible. Utilizing broad and dedicated approaches to investigate multilayered cellular communication offers a new layer of characterization in cancer biology, with potential to enhance both diagnostic precision and therapeutic strategies.

## Conflict of interest

The authors declare no conflict of interest.

## Author contributions

RB and MC conceived and wrote the manuscript.
